# Nurses’ perceptions about the patient safety climate in Primary Health Care

**DOI:** 10.1590/1518-8345.6374.4092

**Published:** 2024-01-26

**Authors:** Edinêis de Brito Guirardello, Mariana Véo Nery de Jesus, Lilian Ceroni Vieira, Henrique Ceretta Oliveira, Maria Silvia Teixeira Giacomasso Vergilio

**Affiliations:** 1 Universidade Estadual de Campinas, Faculdade de Enfermagem, Campinas, SP, Brazil.; 2 Scholarship holder at the Coordenação de Aperfeiçoamento de Pessoal de Nível Superior (CAPES), Brazil.

**Keywords:** Patient Safety, Organizational Culture, Leadership, Nurses, Primary Health Care, Health Evaluation, Seguridad del Paciente, Cultura Organizacional, Liderazgo, Enfermeras y Enfermeros, Atención Primaria de Salud, Evaluación en Salud, Segurança do Paciente, Cultura Organizacional, Liderança, Enfermeiras e Enfermeiros, Atenção Primária à Saúde, Avaliação em Saúde

## Abstract

**Objective:**

to evaluate the patient safety climate in Primary Health Care from the perspective of nurses working in the services.

**Method:**

a quantitative and cross-sectional study conducted with 148 nurses from a municipality in the state of São Paulo. The Brazilian version of the Primary Care Safety Questionnaire Survey and personal, professional, and organizational performance variables (intention to stay at work, job satisfaction, care quality, and frequency of incidents) were used. Parametric and non-parametric comparison tests and Spearman’s correlation coefficient were performed, considering a 5% significance level.

**Results:**

the safety climate was positive, varying from 4.52 to 5.33 and differing across districts for workload (p=0.0214) and leadership (p=0.0129). The safety climate professional variables and dimensions differed in relation to the frequency of incidents. Teamwork and safety and learning system were strongly correlated with job satisfaction and moderately with perceived care quality.

**Conclusion:**

teamwork and safety and learning system stood out for their positive correlations with job satisfaction and care quality. A positive safety climate favors the involvement of Primary Care nurses to develop improvement plans aligned with the National Patient Safety Program.

## Resumen

**Objetivo:**

evaluar el clima de seguridad del paciente en la Atención Primaria de la Salud, desde la perspectiva de los enfermeros de los servicios.

**Método:**

estudio cuantitativo y transversal, con 148 enfermeros de un municipio del Estado de São Paulo. Se utilizó la versión brasileña del *Primary Care Safety Questionnaire Survey* y variables personales, profesionales y de desempeño organizacional (intención de permanecer en el empleo, satisfacción laboral, calidad de la atención y frecuencia de incidentes). Se realizaron pruebas de comparación paramétricas y no paramétricas y coeficiente de correlación de Spearman, considerando un nivel de significación del 5%.

**Resultados:**

el clima de seguridad fue positivo, osciló entre 4,52 y 5,33; difirió entre distritos en carga de trabajo (p=0,0214) y liderazgo (p=0,0129). Las variables profesionales y dimensiones del clima de seguridad difirieron en la frecuencia de incidentes. El trabajo en equipo, el sistema de seguridad y el aprendizaje tuvieron una correlación alta con la satisfacción laboral y una moderada con la percepción de la calidad de la atención.

**Conclusión:**

el trabajo en equipo y el sistema de seguridad y aprendizaje se destacaron por su correlación positiva con la satisfacción laboral y la calidad de la atención. Un clima de seguridad positivo favorece la participación de los enfermeros de la atención primaria en el desarrollo de planes de mejora que sigan las indicaciones del Programa Nacional de Seguridad del Paciente.

## Resumo

**Objetivo:**

avaliar o clima de segurança do paciente na Atenção Primária à Saúde, sob a perspectiva dos enfermeiros dos serviços.

**Método:**

estudo quantitativo e transversal, com 148 enfermeiros de um município do Estado de São Paulo. Foram utilizadas a versão brasileira do *Primary Care Safety Questionnaire Survey* e variáveis pessoais, profissionais e desempenho organizacional (intenção permanecer no trabalho, satisfação no trabalho, qualidade do cuidado e frequência de incidentes). Realizaram-se testes de comparação paramétricos, não paramétricos e coeficiente de correlação de

**Resultados:**

o clima de segurança foi positivo, com variação de 4,52 a 5,33; diferiu entre distritos para carga de trabalho (p=0,0214) e liderança (p=0,0129). As variáveis profissionais e dimensões do clima de segurança diferiram em relação à frequência de incidentes. Trabalho em equipe, sistema de segurança e aprendizagem foram fortemente correlacionados à satisfação no trabalho e moderadamente com percepção da qualidade do cuidado.

**Conclusão:**

trabalho em equipe e sistema de segurança e aprendizagem se destacaram pela correlação positiva com satisfação no trabalho e qualidade do cuidado. O clima de segurança positivo favorece o envolvimento dos enfermeiros da atenção primária para desenvolver planos de melhorias alinhados ao Programa Nacional de Segurança do Paciente.

**Highlights**

**(1)** The safety climate is perceived differently across health districts.

**(2)** There is a correlation between the climate dimensions and professional satisfaction.

**(3)** Workload and leadership exert an influence on the safety climate perception.

**(4)** There is a relationship between the safety climate and reporting of care-related incidents.

**(5)** The safety climate is perceived differently among nurses regarding their role.

## Introduction

Patient safety is defined as a structure of organized activities that creates cultures, processes, procedures, behaviors, technologies and environments conducive to health care that consistently and sustainably reduce risks and the occurrence of preventable harms ^(^ 1 ^)^. Care quality at all health care levels ^(^ 1 ^-^ 2 ^)^ is influenced by Primary Health Care (PHC), which is considered the main gateway and communication center of the Health Care Network ( *Rede de Atenção à Saúde*, RAS) belonging to the Unified Health System ( *Sistema Único de Saúde*, SUS). 

In PHC, the adoption of patient safety precepts is still incipient despite the launch of the National Patient Safety Program ( *Programa Nacional de Segurança do Paciente*, PNSP) in 2013, which established the obligation to implement protocols and strategies guiding actions for safe care in all health services of the country. In 2017, the National Primary Care Policy ( *Política Nacional de Atenção Básica*, PNAB) contributed advances in this theme with a focus on reducing risks and adverse events in PHC services ^(^ 3 ^-^ 4 ^)^. The term “patient safety” can be strange to PHC professionals since, according to the SUS principles, citizens are understood as protagonists and participants in their care, being called users. However, it is worth noting that it is a taxonomy recognized worldwide for all health services. 

Harms to the user arising from unsafe care are therefore a global challenge for managers and public health, as they can cause irreversible disabilities and lead to death ^(^ 1 ^-^ 2 ^)^. In England, the incidence of harms in PHC was from 35.6 to 57.9 per 100,000 users a year and, for the most part, it was related to problems with diagnosis, followed by issues with drug prescription, and a smaller portion with late referrals for treatments, such that the authors highlighted that most of the incidents can be avoided ^(^ 5 ^)^. 

In Brazil, studies on patient safety in PHC indicate that the most common incidents were associated with diagnostic and medication errors, and the contributing factors to these incidents were as follows: failures in care, management and communication with users, with the team and with other RAS levels ^(^ 6 ^)^. In addition to that, they were also associated with administrative errors such as failures involving medical records, incomplete records, loss, misplacement and exchange between users ^(^ 7 ^)^. In addition, personal, organizational and work environment factors, both internal and external, can also influence a negative safety culture ^(^ 8 ^)^. 

Creating a sustainable culture that promotes patient safety is a key condition for reducing adverse events in health institutions, as it makes it possible to find structural and systemic weaknesses and, based on them, take action to improve health ^(^ 1 ^)^. A strong safety culture is fundamental to reducing user harm and providing a safe working environment for health workers ^(^ 1 ^)^. 

The safety climate is an indirect measure of an organization’s safety culture ^(^ 9 ^)^, which involves people’s perspectives and beliefs regarding safety policy and services, organizational attitudes, safety supervision and management ^(^ 10 ^)^. The safety climate assessment through specific instruments is an efficient method for diagnosing aspects of the institutional safety culture ^(^ 11 ^)^, which allows strengthening management in terms of planning actions in this direction ^(^ 12 ^)^. 

The PHC guidelines are to provide a comprehensive, welcoming, safe and responsive service to the health demands of people, families and communities ^(^ 3 ^,^ 13 ^-^ 14 ^)^. Nurses are professionals who play a central role in the management of teams working in PHC services, through their leadership both in technical/care activities and in the planning and coordination of programs established by laws, statutes and guidelines of the Ministry of Health ( *Ministério da Saúde*, MS) and the professional council ^(^ 3 ^,^ 14 ^-^ 15 ^)^. 

It is noted that effective leadership is fundamental to establishing an institutional culture focused on patient safety and understanding that there may be flaws and potential errors in the care production process, which need to be evaluated and corrected ^(^ 16 ^-^ 18 ^)^. In view of the above, in this study the objective was to evaluate the patient safety climate in PHC from the perspective of nurses working in the services. 

## Method

### Type of study

A quantitative and cross-sectional study following the recommendations outlined in STrengthening the Reporting of Observational studies in Epidemiology (STROBE) ^(^ 19 ^)^. It is noted that the patient safety climate in the PHC context will be analyzed from the nurses’ perspective, based on personal, professional, and organizational performance variables. 

### Locus

The study was carried out in a large municipality with an estimated population of 1,236,885 inhabitants, located in the inland of the state of São Paulo, Brazil. This municipality has 66 Health Centers (HCs) distributed across five Health Districts (HDs): North with 12 HCs; South with 17 HCs; East with ten HCs; Northwest with 14 HCs; and Southwest with 13 HCs, with coordination organized in territories with approximately 200,000 inhabitants. Each HC serves nearly 20,000 inhabitants and is managed by a coordinator whose health institution includes from two to five family health teams, according to the population of the area covered by the territory ^(^ 20 ^)^. 

### Period

Data collection was carried out from September 2019 to March 2020.

### Population

The total population corresponded to 249 nurses: 41 from the East District, 52 from the Northwest District, 44 from the North District, 48 from the Southwest District, and 64 from the South District, coming from the different HCs of all five HDs in the municipality.

### Selection criteria

Nurses who worked in coordination and assistance of the HCs and with a minimum experience of six months were considered. Professionals absent due to vacation or other leaves of absence were excluded.

### Sample

The sample size was determined considering the sample calculation methodology to estimate a proportion ^(^ 21 ^)^. The calculation assumed a proportion equal to 0.50, representing the maximum variability of the binomial distribution, 5% sampling error, 5% significance level, and a population of 249 nurses. The calculation resulted in a minimum sample of 151 participants. 

The sample was divided proportionally across the districts according to the population of nurses in each district, with 25 in the East District, 31 in the Northwest District, 27 in the North District, 29 in the Southwest District, and 39 in the South District. The participants were selected through a cluster sampling scheme and stratified according to districts.

The numbers of HCs that made up the study were as follows: ten in the North District; 14 in the South; eight in the East; 11 in the Northwest; and 12 in the Southwest. It is worth noting data collection interruption due to the COVID-19 pandemic, especially with regard to the East District (the last collection site) due to the lack of access to the HCs and the unavailability of nurses. Thus, eventually, it was possible to obtain a sample comprised of 148 nurses: 16 in the East District (out of the 25 planned); 31 in the Northwest; 30 in the North (out of the 27 planned); 32 in the Southwest (out of the 29 planned); and 39 in the South. Therefore, the number of participants from the North and Southwest Districts exceeded the predicted proportional sample.

### Variables

Personal variables of age, gender and marital status were considered. The professional variables were the following: experience time in the current team and PHC; role in the HC in relation to coordination or assistance position; number of teams in the HC; type of team in which they worked (Family Health Strategy [FHS] or Basic Health Unit [BHU] and Community Health Agents Strategy [ *Estratégia de AgentesComunitários de Saúde*, EACS]); whether the team was complete; and whether the professionals had another employment contract. It is worth highlighting that the teams mentioned are organized by the PNAB ^(^ 3 ^)^. 

The safety climate variable was extracted from the Brazilian version of the Primary Care Safety Questionnaire (PC-SafeQuest) ^(^ 22 ^)^ and aims to assesss the user’s perception of the safety climate in PHC. It consists of 28 items distributed across five dimensions: workload (three items) shows impaired performance due to excessive workload, inadequate staffing, time constraints, and the professionals’ expectations when working under pressure; communication (five items) covers the degree to which discussions between team members are open and honest, and whether the professionals feel free to question management decisions; leadership (five items) assesses whether the leaders are open to suggestions for improvements and attitudes towards formal rules and procedures; teamwork (seven items) refers to the perception of its importance and the level of mutual respect and support within teams; and safety and learning system (eight items) assesses the degree to which the practices encourage communication of significant events and the existence of procedures to prevent them ^(^ 23 ^)^. 

These dimensions are evaluated using a Likert scale with the following options: “Not at all (1 point)”; “To a very limited extent (2 points)”; “To a limited extent (3 points)”; “Moderately (4 points)”; “To a considerable extent (5 points)”; “To a significant extent (6 points)”; and “Completely (7 points)”. The score is calculated by the mean value of the answers to each item, and the higher the score, the more positive the professional’s perception of the safety climate. For the current study, a positive safety climate was considered when the mean scores were equal to or greater than four points; and mean scores lower than four classified as a negative safety climate. PC-SafeQuest is one of the PHC climate assessment instruments recognized for its practicality, acceptability, and possibility of identifying weak points that deserve to be investigated and modified ^(^ 11 ^)^. 

The organizational performance variables assessed were as follows: the professional’s intention to stay in the job the following year; perception about the care quality offered to the users in their work environment; job satisfaction; and healthcare-related incidents. The first two variables were evaluated on a scale that varies from zero to ten points, and the closer to ten, the better the perception of care quality and the greater the intention to stay on the job.

The “professional satisfaction” variable about their current position at work was measured using the Job satisfaction subscale, extracted from the Brazilian version of the 2006 Safety Attitudes Questionnaire (SAQ) – Short form ^(^ 24 ^)^. It is noted that this subscale consists of five items, namely: 1. I like my job; 2. Working here is like being part of a big family; 3. This is a good place to work; 4. I am proud to work in this area; and 5. Morale (state of mind/mood) in this area is high. 

These items were evaluated using a Likert scale with the following options: “I totally disagree” (zero points); “I partially disagree” (25 points); “Neutral” (50 points); “I partially agree” (75 points); “I totally agree” (100 points); and the “Not applicable” option for items without a score. The score for each domain is obtained by adding the scores and dividing by the number of questions answered, excluding those with a “Not applicable” answer. Values equal to or above 75 points represent satisfied professionals at work.

Finally, the “healthcare-related incidents” variable covered the following: a) failure to identify the user in procedures and exams; b) failure to identify the user in the consultation and medical records; c) non-adherence to hand hygiene; and d) failure in communication between professionals and users. Any deviation in care that poses a risk of harm to the patient, such as avoidable errors, events, or dangers, were considered incidents ^(^ 14 ^)^. The participants were asked to report the frequency of these incidents in their work unit during the last month, with the following answer options: “Never” (1 point); “Rarely” (2 points); “Frequently” (3 points); and “Very frequently” (4 points). 

### Data collection

One of the authors carried out the collection in person at the HCs. After accepting the invitation, the nurses who met the inclusion criteria to participate in the study received an envelope containing the instruments, two printed copies of the Free and Informed Consent Form (FICF), a pen and a seal. Each nurse agreed on the best day and time to answer the instruments. They also agreed to return them if they could not answer them at that moment. The instruments were self-answered, with the author only available for possible doubts.

The collection was carried out using the Brazilian version of PC-SafeQuest ^(^ 22 ^)^, the Job Satisfaction subscale extracted from the Brazilian version of the Safety Attitudes Questionnaire(SAQ) ^(^ 24 ^)^, and a form with personal and professional variables, organizational performance and frequency of incidents, previously prepared and subjected to content validity by a group of judges. 

### Data analysis

The data collected were entered into an Excel spreadsheet, with double-checking and validation of the database, and analyzed using the Statistical Analysis System (SAS) software, version 9.4, and the Statistical Package for the Social Sciences (SPSS), version 23. A 5% significance level was assumed in the analyses. Descriptive statistics were performed by calculating absolute and relative frequencies for the qualitative variables and of central tendency and dispersion measures for the quantitative ones.

The ANOVA model was applied to analyze the comparisons between districts regarding the PC-SafeQuest scores, followed by Tukey’s post-test or the Kruskal-Wallis test, followed by Dunn’s post-test, according to data distribution. The comparisons considering gender, marital status, role in the HC, and team composition in relation to the PC-SafeQuest scores and between the categories for frequency of occurrence of incidents related to the quantitative variables were carried out using the Student’s unpaired t test or the Mann-Whitney test, according to data distribution. In these analyses, the frequency of incidents reported by the nurses was grouped into the “Never/Rarely” and “Frequently/Very frequently” answer options. Data distribution was assessed using the Shapiro-Wilk test, and variances homogeneity by Levene’s test.

For the correlation analyses between the PC-SafeQuest scores and the other quantitative variables, Spearman’s correlation coefficient was applied, with values from zero to 0.29 considered as weak in magnitude, from 0.30 to 0.49 as moderate in magnitude, and values equal to or above 0.50 as of strong magnitude ^(^ 25 ^)^. 

### Ethical aspects

The institution’s Research Ethics Committee approved the study under Opinion No. 3,179,811, by the recommendations set forth in Resolution No. 466/12 of the National Research Ethics Council.

## Results

The sample included 148 nurses with a mean age of 38.81 years old (SD=7.98), mean experience in PHC of 9.81 years (SD=7.43), and 5.65 years (SD=4.14) in the current team. Table 1 presents other characteristics. 

**Table 1 -:** Characterization of the health centers and nurses by health district (n ^*^ = 148). São Paulo, Brazil, 2020

**Variables**	**Total Sample**		**South District**		**North District**		**Southwest District**		**Northwest District**		**East District**	
	n *	**%** ^†^	n *	**%** ^†^	n *	**%** ^†^	n *	**%** ^†^	n *	**%** ^†^	n *	**%** ^†^
**Nurses**	148	100.00	39	26.35	30	20.27	32	21.62	31	20.95	16	10.81
**Gender**																	
Female	125	84.46	37	94.87	24	80.00	25	78.13	27	87.10	12	75.0
Male	23	15.54	2	5.13	6	20.00	7	21.87	4	12.90	4	25.0
**Marital status**																	
Single	29	19.73	6	15.38	8	26.67	6	18.75	8	25.81	1	6.67
Married/Stable union	101	68.71	29	74.36	19	63.33	22	68.75	20	64.52	11	73.33
Separated/Divorced/Widowed	17	11.56	4	10.26	3	10.00	4	12.50	3	9.68	3	20.00
No information	1		0		0		0		0		1	
**Employment contract**																	
1	136	91.89	38	97.44	28	93.33	29	90.63	27	87.10	14	87.50
2 or more	12	8.11	1	2.56	2	6.67	3	9.38	4	12.90	2	12.50
**Role in the Health** **Center**																	
Coordination	26	17.57	6	15.38	6	20.00	5	15.63	7	22.58	2	12.50
Assistance	122	82.43	33	84.61	24	80.00	27	84.37	24	77.42	14	87.50
**Type of team**																	
FHS [Table-fn tfn3b] ^‡^	119	84.41	30	81.08	28	93.33	27	93.10	19	65.51	15	93.75
EACS ^§^ and BHU ^||^	22	15.60	7	18.91	2	6.67	2	6.89	10	34.48	1	6.25
No information	7		2		0		3		2		0	
**Number of teams**																	
1	13	8.78	1	2.56	2	6.67	2	6.25	8	25.81	0	0.00
2	37	25.00	18	46.15	2	6.67	2	6.25	9	29.03	6	37.50
3	34	22.97	5	12.82	10	33.33	10	31.25	9	29.03	0	0.00
4	54	36.49	15	38.46	16	53.33	17	53.13	1	3.23	5	31.25
5	10	6.76	0	0.00	0	0.00	1	3.13	4	12.90	5	31.25
**Complete Team**																	
Yes	50	35.21	16	44.44	16	53.33	10	33.33	4	13.33	4	25.0
No	92	64.79	20	55.55	14	46.67	20	66.66	26	86.66	12	75.0
No information	6		3		0		2		1		0	

^*^ n = Sample;

^†^ % = Percentage;

^‡^ FHS = Family Health Strategy;

^§^ EACS = *Estratégia de Agentes Comunitários de Saúde* (Community Health Agents Strategy);

^||^ BHU = Basic Health Unit

Regarding the organizational performance variables, the nurses reported a mean score of 75.29 (SD=21.03) for job satisfaction, 8.08 (SD=2.91) for intention to stay on the job, and 7.68 (SD=1.38) for perception about the care quality offered to the users.

As for the perception of the safety climate, the mean scores for the dimensions were as follows: workload (M=4.52; SD=1.06); communication (M=5.33; SD=1.19); leadership (M=5.20; SD=1.25); teamwork (M=5.08; SD=0.97); safety and learning system (M=5.12; SD=1.16); and 5.10 (SD=0.91) for the total score.

In the comparison analyses between the PC-SafeQuest dimensions and personal (gender, marital status) and professional (role in the HC and team completeness) variables, it was verified that they differ in terms of role and completeness of the team. Nurses in the role of coordinators reported higher scores for all PC-SafeQuest dimensions when compared to clinical nurses, with statistically significant differences for the communication (p=0.0093), teamwork (p=0.0116) and safety and learning system (p=0.0030) dimensions and the total score (p=0.0035).

In turn, when comparing PC-SafeQuest to the completeness of the team, it was verified that those who reported working with an incomplete team presented higher scores for the leadership (p=0.0111) and teamwork (p=0.0163) dimensions when compared to those who reported working with a complete team.

Regarding nurses’ perception of the safety climate across the HDs, there were significant statistical differences for the workload and leadership dimensions and total score, as shown in Table 2. 

Regarding the frequency of healthcare-related incidents, the nurses reported 9.52% of frequent or very frequent occurrences for failure to identify the user in procedures and exams, 23.29% for failure to identify the user in consultations and medical records, 29.73% for non-adherence to hand hygiene and 64.19% for failure in communication between professionals and users. The comparisons in relation to the personal and professional variables, care quality, job satisfaction, and PC-SafeQuest dimensions with the frequency of incidents are presented in Table 3. 

Another aspect analyzed in the current study refers to the assessment of the existence of a correlation between the PC-SafeQuest dimensions and personal, professional, and organizational performance variables, which resulted in correlations of strong, moderate, and weak magnitude ( Table 4). 

**Table 2 -:** Comparison of the nurses’ perception of the safety climate across health districts (n * = 148). São Paulo, Brazil, 2020

**PC-SafeQuest ^†^**	**Total** **Sample**		**South** **District**		**North** **District**		**Southwest** **District**		**Northwest** **District**		**East** **District**		**p-value ^||^**
	**Mean/** **Median**	**SD ^‡^/** **IQR ^§^**	**Mean/** **Median**	**SD ^‡^/** **IQR ^§^**	**Mean/** **Median**	**SD ^‡^/** **IQR ^§^**	**Mean/** **Median**	**SD ^‡^/** **IQR ^§^**	**Mean/** **Median**	**SD ^‡^/** **IQR ^§^**	**Mean/** **Median**	**SD ^‡^/** **IQR ^§^**	
Workload	4.52	1.06	4.53	0.98	4.18 ^¶^	0.91	4.52	1.15	5.01 ^¶^	1.13	4.19	0.94	**0.0214** ^**^
Communication	5.60	1.60	5.20	1.60	5.30	1.40	5.90	1.80	6.00	1.20	5.10	1.30	0.0746 ^††^
Leadership	5.40	2.00	4.80 ^‡‡^ ^§§^	2.00	5.40	2.00	5.90 ^‡‡^	2.20	5.80 ^§§^	1.60	5.00	1.50	**0.0129** ^††^
Teamwork	5.29	1.57	5.14	1.42	5.07	1.28	5.43	1.22	5.71	1.43	5.14	1.50	0.0889 ^††^
Safety and learning system	5.38	1.87	5.13	2.00	4.88	1.50	5.63	1.19	5.63	1.75	5.13	2.62	0.1399 ^††^
Total score	5.22	1.34	5.04	1.29	4.88	1.04	5.46	1.55	5.68	1.33	5.00	1.25	**0.0250** ^††^

^*^ n = Sample;

^†^ PC-SafeQuest = Primary Care Safety Questionnaire;

^‡^ SD = Standard Deviation;

^§^ IQR = Interquartile range;

^||^ p-value = Significance probability;

^¶^ Tukey’s post-test: North x Northwest were significant;

^**^ ANOVA test – Comparisons based on mean and standard deviation;

^††^ Kruskal-Wallis test – Comparisons based on the median and IQR;

^‡‡^ Dunn’s post-test: South x Southwest were significant;

^**§§^ Dunn’s post-test: South x Northwest were significant

**Table 3 -:** Comparison between personal and professional variables, care quality, job satisfaction, PC-SafeQuest ^*^ dimensions and frequency of incidents (n ^†^ = 148). São Paulo, Brazil, 2020

**Variables**	**Frequency**	**Incident A ^‡^**			**Incident B ^§^**			**Incident C ^||^**			**Incident D ^¶^**		
		**Mean/** **Median**	SD ^**^/ **IQR ^††^**	**p-value ^‡‡^**	**Mean/** **Median**	SD ^**^/ **IQR ^††^**	**p-value ^‡‡^**	**Mean/** **Median**	SD ^**^/ **IQR ^††^**	**p-value ^‡‡^**	**Mean/** **Median**	SD ^**^/ **IQR ^††^**	**p-value ^‡‡^**
Age	1 ^§§^	36.00	9.00	0.0949 ^||||^	36.00	10.50	0.5540 ^||||^	36.00	9.50	0.6787 ^||||^	37.00	15.00	0.3943 ^||||^
	2 ^¶¶^	41.50	12.00		36.00	8.00		36.50	11.50		36.00	9.00	
Experience time in the current team	1 ^§§^	4.75	4.92	0.5698 ^||||^	4.71	4.62	0.7454 ^||||^	5.00	4.58	**0.0473** ^||||^	5.00	5.00	0.9186 ^||||^
	2 ^¶¶^	4.00	5.66		4.46	5.00		4.08	2.12		4.58	4.75	
Experience time in Primary Care	1 ^§§^	8.16	5.17	0.8818 ^||||^	8.62	6.00	**0.0427** ^||||^	8.83	6.50	**0.0007** ^||||^	9.00	13.00	0.0681 ^||||^
	2 ^¶¶^	8.04	8.00		6.88	5.00		5.00	5.37		8.00	4.75	
Intention to stay in the job	1 ^§§^	10.00	2.00	**0.0237** ^||||^	10.00	2.00	**0.0084** ^||||^	10.00	2.00	0.2143 ^||||^	10.00	2.00	0.3881 ^||||^
	2 ^¶¶^	7.50	8.00		8.00	5.00		9.00	4.00		9.00	3.00	
Perception about care quality	1 ^§§^	8.00	1.00	0.1332 ^||||^	8.00	2.00	**0.0068** ^||||^	8.00	2.00	**0.0011** ^||||^	8.00	1.00	**<0.0001** ^||||^
	2 ^¶¶^	7.50	3.00		7.00	2.00		7.00	2.00		8.00	1.00	
Job satisfaction	1 ^§§^	80.00	25.00	0.4494 ^||||^	80.00	22.50	**0.0401** ^||||^	82.50	25.00	0.0521 ^||||^	85.00	15.00	**<0.0001** ^||||^
	2 ^¶¶^	80.00	15.00		75.00	45.00		77.50	30.00		75.00	30.00	
Workload	1 ^§§^	4.54	1.08	0.8335 ^||||^	4.67	1.17	0.7622 ^||||^	4.59	1.09	0.2020 ^***^	4.67	1.33	**0.0110** ^||||^
	2 ^¶¶^	4.48	0.84		4.50	1.66		4.35	0.99		4.33	1.66	
Communication	1 ^§§^	5.60	1.40	0.5496 ^||||^	5.60	1.40	0.3186 ^||||^	5.60	1.40	0.3326 ^||||^	5.80	1.20	**0.0002** ^||||^
	2 ^¶¶^	5.00	1.60		5.20	2.00		5.50	1.70		5.20	1.80	
Leadership	1 ^§§^	5.40	2.00	0.7662 ^||||^	5.40	1.80	**0.0176** ^||||^	5.40	1.80	0.1472 ^||||^	5.80	1.40	**0.0107** ^||||^
	2 ^¶¶^	5.40	2.20		4.80	2.00		5.00	2.10		5.00	2.20	
Teamwork	1 ^§§^	5.29	1.57	0.9763 ^||||^	5.36	1.29	**0.0365** ^||||^	5.43	1.57	0.0702 ^||||^	5.71	1.00	**<0.0001** ^||||^
	2 ^¶¶^	5.29	0.71		4.71	2.00		5.14	1.14		5.00	1.43	
Safety and learning system	1 ^§§^	5.50	1.87	0.3673 ^||||^	5.56	1.38	**0.0002** ^||||^	5.50	1.75	0.0856 ^||||^	5.75	1.00	**0.0002** ^||||^
	2 ^¶¶^	5.00	1.75		4.25	2.13		4.94	1.94		4.88	1.88	
Total score	1 ^§§^	5.25	1.33	0.7842 ^||||^	5.36	1.31	**0.0036** ^||||^	5.36	1.45	0.0652 ^||||^	5.51	0.79	**<0.0001** ^***^
	2 ^¶¶^	4.88	1.25		4.55	1.65		4.93	1.34		4.86	0.89	

^*^ PC-SafeQuest = Primary Care Safety Questionnaire;

^†^ n = Sample;

^‡^ Failure to identify the user in procedures and exams;

^§^ Failure to identify the user in the consultation and medical records;

^||^ Non-adherence to hand hygiene;

^¶^ Failure in communication between professionals and users;

^**^ SD = Standard Deviation;

^††^ IQR = Interquartile Range;

^‡‡^ p-value = Significance probability;

^§§^ Never/Rarely;

^||||^ Mann-Whitney test – Comparisons based on the median and IQR;

^¶¶^ Frequently/Very frequently;

^***^ Unpaired Student’s t test – Comparisons based on mean and standard deviation

**Table 4 -:** Spearman’s correlation coefficients between the PC-SafeQuest ^*^ dimensions and personal, professional, and organizational performance variables (n ^†^ = 148). São Paulo, Brazil, 2020

PC-SafeQuest ^*^	**Age**	**Time in PHC ^‡^**	**Number of teams in the HC ^§^**	**Intention to staying the job**	**Care quality**	**Job satisfaction**
Workload	0.0427	-0.0436	-0.2209 ^||^	0.0846	0.3532 ^||^	0.2969 ^||^
Communication	0.0082	-0.0995	0.0239	0.1417	0.2834 ^||^	0.4395 ^||^
Leadership	0.1448	0.0284	-0.0250	0.2062 ^||^	0.3187 ^||^	0.4376 ^||^
Teamwork	0.1102	-0.0116	-0.0454	0.2484 ^||^	0.3957 ^||^	0.6444 ^||^
Safety and learning system	0.1813 ^||^	0.0889	-0.0588	0.2278 ^||^	0.4324 ^||^	0.5052 ^||^
Total score	0.1419	0.0112	-0.0663	0.2468 ^||^	0.4401 ^||^	0.5826 ^||^

^*^ PC-SafeQuest **=** Primary Care Safety Questionnaire;

^†^ n = Sample;

^‡^ Primary Health Care;

^§^ HC = Health Center;

^||^ p-value<0.05

## Discussion

The sample of nurses in this study comprised young adults, most of whom were women and responsible for care activities in the different centers and HDs. The nurses’ extensive experience in PHC and the current team, coupled with only one employment contract, indicates that they are skilled professionals who are duly prepared and committed to performing their activities. With time in service, the professionals can understand the development of their work, available resources, and interactions with the work team in a collaborative process responsible for delivering safe and efficient care ^(^ 2 ^)^. 

In this study, a certain mismatch in the availability of human resources was identified, so the majority answered that their work team was not complete; however, they reported offering good care quality to the users, job satisfaction, and intention to stay on the job. On the other hand, a study carried out in Spain with PHC nurses also highlighted problems related to the adequacy of human resources, such as an insufficient number of professionals to carry out the work, insufficient time and opportunities to discuss care-related issues, and insufficient support services that allow nurses to devote more time to the users, which were highlighted as weaknesses in the nurses’ practice environment in PHC to ensure the care quality provided ^(^ 26 ^)^. 

The nurses reported a positive perception of the safety climate, whose values are close to studies carried out in the United Kingdom ^(^ 9 ^,^ 27 ^-^ 28 ^)^ and Ireland ^(^ 29 ^)^, which used the same instrument. These are compared to studies carried out with PHC health professionals using other instruments, such as the Medical Office Survey on Patient Safety Culture in Greece ^(^ 2 ^)^ and in Kuwait ^(^ 30 ^)^ and the Hospital Survey on Patient Safety Culture in Oman ^(^ 17 ^)^. 

The favorable safety climate indicates that the professionals perceive that the coordination of the units is focused on safeguarding the care procedures and the professionals’ safety through clear, participative communication and actions aligned with an institutional policy devoted to safety and quality ^(^ 31 ^)^. This study identified statistically significant differences in the perception of the safety climate between coordinating nurses and those responsible for user assistance activities, in which the coordinators attributed higher scores to the communication, teamwork, safety, and learning system dimensions and the PC-SafeQuest total score. 

These dimensions were also evaluated in a study in England ^(^ 9 ^)^ with PC-SafeQuest, showing that managers classified the safety climate as significantly more positive than non-managers. The same is true with another study in Scotland ^(^ 27 ^)^, which obtained statistically significant differences in the perception of the safety climate among management professionals in relation to other workers. Both studies conclude that the variation in the safety climate perception among certain groups of professionals should be aligned to build a solid safety culture ^(^ 9 ^,^ 27 ^)^. 

An unexpected result in this study was that the nurses who reported working with an incomplete team had more positive perceptions about the leadership and teamwork dimensions when compared to those who working with a complete team. As this is a study with a specific sample of nurses, no other surveys were found for comparison purposes; one of the possible reasons is the fact that, regardless of whether or not they exercise the unit coordinator role, these nurses are team leaders and are able to develop collaborative work with autonomy to prioritize the unit’s service demands. One study highlights that effective leadership is fundamental for developing a safety culture within an organization ^(^ 16 ^)^. 

It is noted that working with an incomplete team can impair patient safety due to work overload ^(^ 18 ^)^. A national study conducted with PHC nurses from several Brazilian regions identified that the professionals in Family Health teams are exposed to physical and mental workloads resulting from excessive demands and insufficient professionals, which can compromise their health and care quality ^(^ 32 ^)^. A study in England highlights the relationship between increased workload and exposure to stress among health professionals involved in clinical practice and management ^(^ 9 ^)^. 

The workload and leadership dimensions differed between nurses in the different HDs, where those from the Northwest District have a more positive perception of workload when compared to nurses from the North District. Although the Northwest District is responsible for assisting users in a context of greater vulnerability and has greater difficulty retaining PHC professionals due to its geographic location in relation to the North District, it presented a more positive perception of the safety climate in the workload dimension.

Likewise, nurses from the Northwest and Southwest districts have a better perception of leadership when compared to those from the South District. One of the reasons for the negative perception of the safety climate for leadership is that the South District is considered the largest in the municipality in terms of population and PHC services, which constitutes a challenge for leadership actions.

It is noted that the Northwest and Southwest districts are in a territory that has medium- and high-complexity health care services to support SUS professionals and users. Health practices in areas with greater vulnerability and with more users can negatively impact safety, with high workloads and tensions in decision-making, mainly when the region does not have social and health facilities for care continuity ^(^ 33 ^)^. 

Nurses with longer experience in PHC, greater intention to stay in the job, more satisfaction at work, and better perceptions about care quality and the safety climate reported lower frequencies of healthcare-related incidents. Although there is no consensus in the literature about these incidents in PHC, several authors emphasize that they should not be associated with those in hospital care. The work guidelines, structure, and dynamics of care differ greatly in PHC, and it becomes crucial to identify incidents reported by professionals based on the experience of their everyday practice ^(^ 7 ^)^. 

A lower frequency of failures to identify the user in procedures and exams was identified, related to nurses’ greater intention to stay on the job, as well as the failure to identify the user in the consultation and medical records, which was also less frequent for nurses who reported job satisfaction, better perception about care quality and longer experience in PHC. Longer experience in the current team and in PHC and a better perception of care quality were also related to fewer failures in adhering to hand hygiene. It was verified that a better perception of quality and satisfaction with work are related to a lower frequency of communication failures between professionals and users.

Correct identification of the users in all service environments circulating within the HC is a basic and indispensable procedure to avoid errors. However, this is not routine in PHC, given that failures in medical records have been reported as contributing factors to errors, especially in FHS units, where the arrangement in the family medical record format, comprised of multiple users, contains flaws in its organization and maintenance due to handling and storage ^(^ 6 ^)^. 

In this study, the results showed that the lower the frequency of failures to identify the user in the consultation and medical records, the more positive the perception of the safety climate by nurses for the leadership, teamwork, and safety and learning system dimensions, although not for the workload and communication dimensions. It is also interesting to note that the lower frequency of communication failures between professionals and users resulted in a more positive perception of the safety climate for all PC-SafeQuest dimensions.

The assessment of correlations between the PC-SafeQuest dimensions and personal and professional variables showed that the safety, learning system, and teamwork dimensions resulted in correlations of strong magnitude with job satisfaction and moderate with the care quality perception. The communication and leadership dimensions resulted in a moderate correlation with job satisfaction. In turn, the leadership and workload dimensions moderately correlated with the perception care quality.

The World Health Organization emphasizes leadership training as one of the factors to ensure improvements in healthcare safety and, therefore, permanent education programs should be valued in health institutions ^(^ 14 ^)^. A study that evaluated the impact of a training program on leadership in patient safety among nurses in the role of manager and clinical nurses in a hospital institution from China resulted in an improvement in nurse-managers’ self-efficacy and leadership behaviors and clinical nurses’ safety behaviors, as well as promoting self-efficacy and safety behaviors and reducing burnout in these latter’s work ^(^ 34 ^)^. 

The importance of studies on this topic in PHC is highlighted in the context of the current health programs and policies, as such services incorporate this prerogative as care coordinators and organizers within the RAS scope.

The results of the current study are motivating due to the novelty of the theme in PHC, in addition to being able to encourage leaders to strengthen the safety culture in such health care points, as well as signaling that coordinators and clinical nurses should value the workload and communication dimensions for the proper functioning of the work done and of the team relationships in the PHC context.

As a limitation, there is data collection interruption in the East District due to the COVID-19 pandemic, which reduces the representativeness of this district. In addition to that, the number of participants from the North and Southwest districts did not follow what had been planned in the sample calculation.

## Conclusion

The perception of the safety climate by nurses in PHC was positive and differed across HDs for the workload and leadership dimensions. The professional variables and dimensions of the safety climate differed in relation to the frequency of incidents, mainly regarding failures in communication between professionals and users, and the identification of the user in the consultation and medical records. The dimensions relevant to teamwork and safety and the learning system showed correlations of strong magnitude with job satisfaction and moderate magnitude with the care quality perception.

Managers, professionals, and users will be able to plan and implement actions to strengthen the dimensions that contribute to a positive safety climate and reevaluate those that require continuous improvement, aligned with the PNSP, with a view to strengthening the safety culture and procedures in PHC. It is recommended that future studies be carried out, given the relevance and scarcity of studies addressing patient safety in PHC.
